# Recurrence quantification analysis to characterize cyclical components of environmental elemental exposures during fetal and postnatal development

**DOI:** 10.1371/journal.pone.0187049

**Published:** 2017-11-07

**Authors:** Paul Curtin, Austen Curtin, Christine Austin, Chris Gennings, Kristiina Tammimies, Sven Bölte, Manish Arora

**Affiliations:** 1 Department of Environmental Medicine and Public Health, Icahn School of Medicine at Mount Sinai, New York City, New York, United States of America; 2 Senator Frank R Lautenberg Environmental Health Sciences Laboratory, Department of Environmental Medicine and Public Health, Division of Environmental Health, Icahn School of Medicine at Mount Sinai, New York City, New York, United States of America; 3 Department of Women’s and Children’s Health, Center of Neurodevelopmental Disorders (KIND), Karolinska Institutet, Stockholm, Sweden; 4 Center for Psychiatry Research, Stockholm County Council, Stockholm, Sweden; University of Tennessee Health Science Center, UNITED STATES

## Abstract

Environmental exposures to essential and toxic elements may alter health trajectories, depending on the timing, intensity, and mixture of exposures. In epidemiologic studies, these factors are typically analyzed as a function of elemental concentrations in biological matrices measured at one or more points in time. Such an approach, however, fails to account for the temporal cyclicity in the metabolism of environmental chemicals, which if perturbed may lead to adverse health outcomes. Here, we conceptualize and apply a non-linear method–recurrence quantification analysis (RQA)–to quantify cyclical components of prenatal and early postnatal exposure profiles for elements essential to normal development, including Zn, Mn, Mg, and Ca, and elements associated with deleterious health effects or narrow tolerance ranges, including Pb, As, and Cr. We found robust evidence of cyclical patterns in the metabolic profiles of nutrient elements, which we validated against randomized twin-surrogate time-series, and further found that nutrient dynamical properties differ from those of Cr, As, and Pb. Furthermore, we extended this approach to provide a novel method of quantifying dynamic interactions between two environmental exposures. To achieve this, we used cross-recurrence quantification analysis (CRQA), and found that elemental nutrient-nutrient interactions differed from those involving toxicants. These rhythmic regulatory interactions, which we characterize in two geographically distinct cohorts, have not previously been uncovered using traditional regression-based approaches, and may provide a critical unit of analysis for environmental and dietary exposures in epidemiological studies.

## Introduction

Exposures to exogenous elements commence early in fetal development and continue throughout life, with marked changes in the routes of exposures and pathways that regulate them. These provide nutrients critical to healthy development, including Ca and Zn, but may also include elements with generally toxic effects or low physiologic tolerance, including Pb, Cr, and As [1–3]. The relevance of exposure timing, intensity, and elemental composition to developmental outcomes is a critical focus in environmental epidemiology, with relevance to the etiology of multiple disease areas and health outcomes.

Elemental exposure biomarkers in blood and urine can provide a window to elemental concentrations at specific points in time, but do not provide a fine-scale temporal history of exposure. This has limited the ability of exposure biologists to study cycles that are inherent in the metabolism of toxic metals and dietary elements. For example, it has been shown by repeated blood sampling at 4 hourly intervals that zinc levels follow a daily cycle characterized by peaks in early morning and markedly lower levels at night [4]. However, it has not been possible to obtain such refined data in large epidemiologic studies, nor to study these rhythms over prolonged periods, using traditional biomarkers. Consequently, environmental epidemiologic methods have largely failed to uncover the cyclical context of endogenous biological and environmental systems. Methods that can characterize the dynamic components of an exposure profile over time are therefore a clear necessity.

The recent development of methods to assay elemental exposures in tooth dentine matrices expands this capacity by allowing continuous longitudinal assessments of elemental concentrations throughout pre- and post-natal development [5,6]. The ebb and flow of elemental concentrations over time can thus be studied to identify critical developmental windows for exposure-related health effects, but may also provide insight into the mechanisms involved in metabolizing elemental exposures. Arora et al. [7] applied a statistical solution, the distributed lag model (DLM), to the challenge of identifying critical developmental windows in longitudinal exposure profiles derived from teeth. This approach allowed a precise specification of developmental windows relevant to a given health outcome, but the fundamental unit of analysis was the elemental concentration at a given time-point. An analytical approach to characterize cyclical components in elemental exposures thus remains elusive.

Analytical methods appropriate for characterizing dynamic periodic components in time series data are abundant in signal processing and physical sciences. Fourier analyses, and wavelet transformations, particularly, are ubiquitous in diverse fields and applications related to time-series analysis. These traditional approaches are nonetheless best-suited to stationary signals with high sampling rates and low noise, and thus poorly suited to an exposures context [8–10].

Recurrence quantification analysis (RQA) [11–13] presents an alternative non-linear method of characterizing signal dynamics that is particularly robust in contexts with short, noisy, and non-stationary signals [8–10, 14–16]. For these reasons, RQA appears in diverse applications in biological fields, including neuroscience, proteomics, cardiology, and behavioral psychology (reviewed in [15–19]). For example, RQA has been used to analyze periodicities in posture and gait [20–22] and eye movement [23, 24]. RQA has also been applied in the analysis of cardiac rhythmicity [25–28], for quantitative assessments of respiratory dynamics [29, 30], and in the analysis of electrophysiological recordings [31–35]. RQA has further proven useful in the analysis of genetic and proteomic sequences [36–40]. Thus, overall RQA is a useful tool for characterizing periodic properties of biological signals at multiple stages of organization.

In the present paper we describe the key concepts underpinning RQA and provide a step-by-step development of the RQA method for an epidemiologic audience. We demonstrate the application of this technique to environmental health studies by using time series data on developmental element exposure profiles. The data we use were generated using laser ablation inductively coupled plasma mass spectrometry (LA-ICP-MS), and tracked pre- and post-natal dentine concentrations of elements. Our analysis reveals significant differences in the cyclical properties of exposure profiles associated with nutrient elements (Zn, Mn, Mg, Ca), as compared to toxicants (Pb, Cr, As). We characterize these effects in two distinct cohorts, with a discovery cohort from Sweden [41] and a replication cohort in the United States, demonstrating both the consistency of these patterns and their broad relevance to human health.

## Materials and methods

### Study samples and laboratory analyses

In our Swedish discovery cohort [41], we studied elemental distribution in the deciduous teeth of 17 females and 25 males who had no known developmental disorders. Our validation cohort in the United States was composed of 12 males and 13 females. Our approach to measuring elements in teeth using laser ablation-inductively coupled plasma mass spectrometry (LA-ICP-MS) and assigning developmental times has been detailed elsewhere [5–7]. The analysis of teeth data in our study received ethical approval from the review board of the Icahn School of Medicine at Mount Sinai, New York (IRB no. IRB-16-00742).

### Recurrence plots

**Fig 1:**
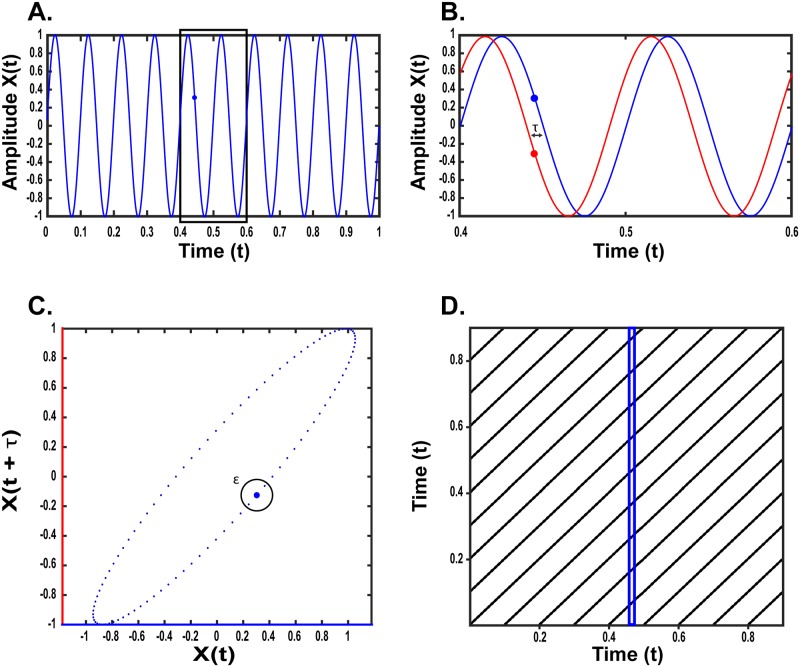
Construction of recurrence plots. A) A 10 Hz sinusoid of wavelength 100 ms. B) In blue, 200 ms of the signal shown in A (indicated by the black box in panel A). Red line is a vector generated by a delay embedding, whereby the original signal is duplicated with a time-lagged delay of interval (*τ*), here 10 ms. C) Phase-portrait of the 10 Hz sinusoid in (A) plotted in two-dimensions (see text). The x-y coordinates of a given point are determined by the value of amplitude of the delayed embedded signals at each sampling interval. Black circle indicates an exemplar threshold (*ε*) used to construct recurrence matrix (D). D) Recurrence plot of 10 Hz sinusoid. Blue rectangle highlights the points captured by the threshold in C (see text). Black points indicate the timing of repetitions as the system returns to a given state, defined by threshold ε.

Recurrence plots (RPs) are a graphical tool for the visualization and analysis of temporal structures in longitudinal experimental data; RPs are additionally critical in the quantitative analysis of temporal structures with recurrence quantification analysis (RQA; see below section). The theoretical basis, development, and application of RPs in diverse research fields are well-described in reviews by Marwan et al. [17–19] and Webber et al. [15,16]. However, these methods have not been used in environmental epidemiologic studies. We therefore provide sufficient details of the theoretical framework underpinning this method for readers to interpret our results. In Fig 1, we provide a graphical outline describing the construction and interpretation of RPs.

Fig 1A illustrates a sinusoidal waveform generated as an exemplar signal for RP analysis. Fig 1B shows the first stage in the construction of an RP analysis, the delay embedding process. With delay embedding the original signal is duplicated but delayed by a given interval of length *τ*, yielding two dimensional vectors (red and blue lines) with dissimilar values at a given index time-point. For simplicity, in this example only a single additional vector is created but this process may yield additional higher dimensions, as well. The number of additional vectors created in the delay embedding process is represented as *m*, a critical parameter in recurrence analysis (see below).

In Fig 1C, the signals yielded from the delay embedding process are used to construct a phase portrait, which takes the range of values in the embedded signals as its axes. For example, if at a given time point the value of the original signal (blue line) is 3 and delayed signal (red line) is 5, then a point is plotted in the phase portrait at coordinates x = 3, y = 5. The full range of values for each vector is iteratively plotted in the construction of the phase portrait, thus capturing the full motion of the system as a function of multiple embeddings. While in this simplified example only two dimensions are plotted, the delay embedding process typically yields additional higher dimensions to allow the construction of 3-dimensional portraits. Because here we embedded a perfectly rhythmic sinusoid, the resultant phase portrait is circular/ellipsoid. The shape of this ellipsoid captures the movement of the system across one period/wavelength, with each subsequent cycle creating overlapping points as the system repeats.

Fig 1D presents the recurrence plot which is the final culmination of this process. The RP is constructed by first determining a threshold value, *ε*, illustrated in Fig 1C as a black circle that is applied to points in the phase portrait. This threshold is iteratively applied to each point in the phase portrait, and a matrix is constructed to capture the index value (timing) of each point that falls within that threshold, thereby capturing the timing of each repetition of the system's movement. This is represented on the RP as a black dot; for example, within the blue-highlighted rectangle on the RP (Fig 1D), every black dot we see represents a point where the system returned to the same value (within-threshold, *ε*) it held at that time-point. With reference to the x-axis and y-axis, the timing of these repetitions can be identified; in this example, with the highlighted rectangle located at approximately 0.5 s on the x-axis, the intervals at which the system enters this state can be identified by aligning recurrence points along the y-axis. This process is iterated for all time points across the signal, with the resultant structures reflecting the periodic properties of the signal.

In this example (Fig 1D), using a perfectly periodic sinusoid, the RP yields a structure composed entirely of diagonal lines, indicative of a system smoothly proceeding in periodic motion. In total 10 lines are apparent, reflecting the 10 cycles of the exemplar signal chosen, and these are each separated vertically by 100 time intervals (ms), reflecting the exemplar signal's wavelength. The features of the RP thus capture the temporal structure of the original signal, and, further, these features are easily amenable to cross-signal qualitative and quantitative comparison. Appendix A and B (in S1 File) show similarly presented examples with a sinusoid injected with random noise, and with an elemental exposure profile like the profiles analyzed in this study. The latter example is additionally embedded in three dimensions, as is typical.

**Fig 2:**
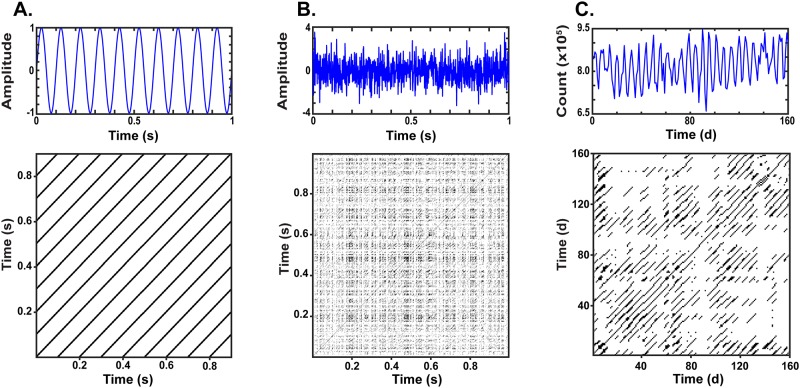
Recurrence plots for periodic, random, and experimental data. A) Top: A 10 Hz sine wave of wavelength 100 ms. Bottom: Recurrence plot (RP) of a 10 Hz sine wave (embedding dimension = 6, time delay = 6, Fixed Recurrence Rate (RR) = 10%. B) Top: Time series of random noise with a mean of 0, and standard deviation of 1. Bottom: RP of random noise (embedding dimension = 6, time delay = 1, Fixed RR = 10%. C) Top: Time series of Ca exposure profile, showing Ca concentration from -89 to 300 days since birth, reflecting 160 samples with an average sampling interval of 2.4 days. Bottom: RP of Ca count (embedding dimension = 4, time delay = 1, Fixed RR = 10%).

In Fig 2, we contrast RPs generated from simulated periodic data (Fig 2A), simulated white noise (Fig 2B), and experimental data reflecting longitudinal measurements of dentine Ca concentrations (Fig 2C). As before, a perfectly periodic system yields a recurrence plot entirely composed of diagonal structures. The randomly generated signal (Fig 2B), in contrast, yields a scattering of recurrence points (black dots) which fail to coalesce in any vertical, horizontal, or diagonal structures, reflecting the absence of temporal organization. The experimental data (Fig 2C), unlike either simulated data series, yields both diagonal elements, indicative of complex (varying in length) and intermittent periodic signal components, and accompanying scattered singular recurrence points. These properties can be further characterized in a quantitative analysis.

The Cross-Recurrence Toolbox package developed by Marwan [42] for Matlab (Mathworks, MA) was used in the construction of RPs, and in the calculation of the critical parameters in the recurrence analysis. The determination of an appropriate delay interval, *τ*, was calculated with a mutual information algorithm, as per the method of Marwan [17, 18]. Similarly, the appropriate number of embedding dimensions, *m*, was determined with a false nearest neighbors (FNN) algorithm. To facilitate comparisons of diverse signals, an adaptive algorithm was used to set threshold (*ε*) values such that recurrence rates (see below section) were fixed to 10% in all plots.

### Recurrence quantification analysis (RQA)

Recurrence quantification analysis (RQA) focuses on derived measures of the principle structural elements evident in RPs; that is, the diagonal, vertical, and horizontal lines formed by successive recurrent points, i.e., the black dots in recurrence plots. Of these, diagonal lines are of principle interest in characterizing periodic components in time-series data, as their distribution and duration indicate the abundance and timing of periodic signal components. Laminar structures, e.g. vertical or horizontal lines, are also of interest as indicators of signal stability, for example when a signal stabilizes at a given intensity for a period of time.

*Mean diagonal length*, consequently, is a critical measure derived from RQA, reflecting a straightforward measurement of the average length of diagonal lines present in a recurrence matrix. This measure can be taken as an absolute indicator of the duration of periodic components in a given signal. *Determinism*, similarly, is derived from analysis of diagonal structures, but here quantifies the relative ratio of diagonal elements to other components in a recurrence matrix, thus indicating the overall periodic content of a given signal. *Recurrence time* captures the mean interval between diagonal elements, while *Shannon entropy* reflects the variability in the distribution of diagonal lengths, with low entropy signals exhibiting little complexity in the distribution of periodic components, and high entropy signals exhibiting diversity in short- and long-duration periodicities.

RQA also captures the extent to which vertical and/or horizontal structures emerge in recurrence matrices, reflecting signal constancy. *Trapping time* describes the mean length of laminar (vertical/horizontal) structures, analogous to how mean diagonal length captures periodic durations. *Laminarity*, an overall measure of signal stability, quantifies the ratio of recurrence points belonging to laminar structures against the total frequency of recurrence points.

The validity of these measures was tested with a twin-surrogate analytical procedure [43–45], whereby the sequential data are randomly shuffled and re-analyzed with RQA, in order to confirm the observed recurrence features are a product of the temporal organization of the data rather than a spurious auto-correlation. From this perspective a meaningful measurement of dynamical features should be statistically different from the measurement of the surrogate time series.

In these analyses, all RPs and RQA analyses were calculated with the Cross-Recurrence Toolbox package [42] in Matlab 2016a (Mathworks, MA).

### Cross-recurrence quantification analysis (CRQA)

**Fig 3:**
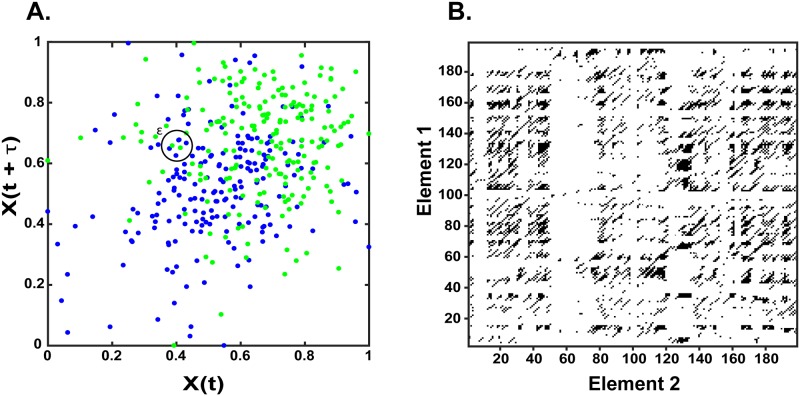
Cross-recurrence plots. A) Phase-portrait of two elements (see text), sampled between -111 to 304 days since birth, plotted in two dimensions. Black circle indicates exemplar threshold (*ε*). B) Cross-recurrence plot of two elements from A.

Recurrence analyses can be extended to multivariate contexts via the implementation of cross-recurrence quantification analysis (CRQA). Fig 3 illustrates this process which closely follows the methods employed in the construction and analysis of RPs. Briefly, two signals in our experiments comprising of the developmental exposure profiles of different elements are simultaneously delay embedded following the procedures outlined in the prior section. These embeddings are then projected in a mutual phase portrait, as in Fig 3A, with different elements represented as green or blue, and a threshold value, *ε*, is iteratively applied to each point to generate a recurrence matrix, as in Fig 3B. Unlike in a singular recurrence analysis, with cross-recurrence the threshold captures when each signal enters the threshold range of the other signal, thus capturing their co-evolution through phase-space.

The recurrence matrix generated in cross-recurrence analysis is amenable to quantitative assessment of diagonal and laminar structural elements via CRQA. In CRQA, however, the interpretation of these measures differs from a univariate analysis, in that the co-evolution of two systems can be determined. Diagonal structures thus represent intervals of synchronous periodicity, while laminar structures reflect shared intervals of signal stability.

As with RQA, all cross-recurrence plots and CRQA analyses were implemented in the Cross-Recurrence Toolbox package [42] in Matlab 2016a (Mathworks, MA).

### Statistical analysis

Measures derived from RQA/CRQA were analyzed in SAS version 9.4 with linear mixed models (PROC MIXED) treating elemental type as a repeated measure across subjects with age and sex as covariates. Post-hoc tests (Tukey) were adjusted for multiple-comparisons. Shapiro-Wilks tests were used to confirm the normal distribution of continuous variables used in linear models.

## Results

### Developmental elemental exposure profiles

**Fig 4:**
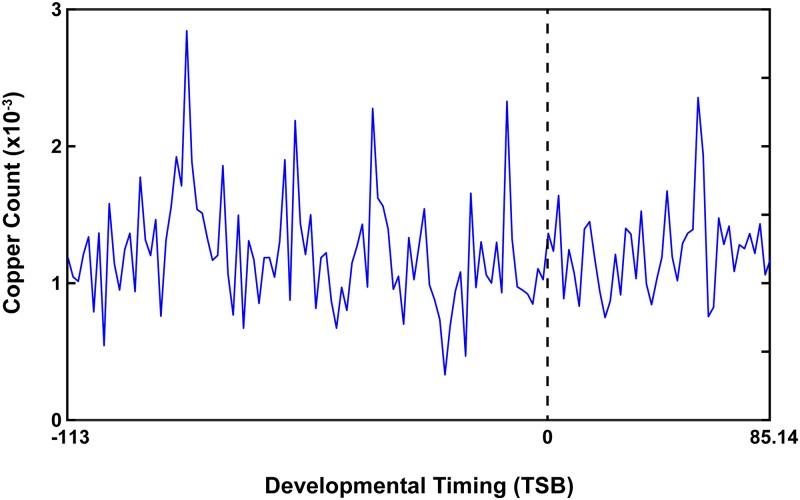
Exemplar elemental exposure profile. In this example, Cu concentration (y-axis) is plotted over time (x-axis). This exposure profile covered a developmental period of 198.14 days, beginning 113 days before birth and ending 85.14 days post-birth. Birth is represented by the 0 point.

In our discovery cohort, we initially measured dentine elemental exposure profiles in 42 teeth covering a mean period of 327.45 days (SD = 107.24), with a mean range of 116.83 (SD = 16.86) days before birth to 210.61 (SD = 110.46) days post-birth. Elements were sampled from teeth at approximately 2 day intervals (M = 2.27, SD = 0.81), with a mean of 147.93 (SD = 23.88) samples per tooth. Fig 4 illustrates the timing parameters of a copper developmental exposure profile for one tooth.

### Recurrence quantification analysis (RQA)

**Fig 5:**
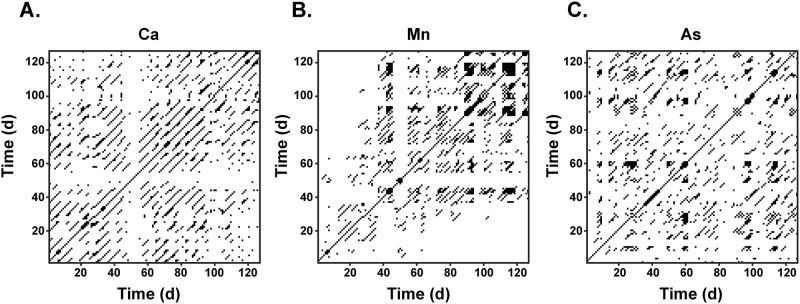
Recurrence plots for elemental exposure profiles. Recurrence plots of three different elements (sampled in the same subject from 11 to 200 days since birth) showing variation in the diagonal, horizontal and vertical recurrence structures. A) Recurrence plot (RP) of Ca time series. B) RP of Mn time series. C) RP of Cr time series.

We began by constructing recurrence matrices to examine temporal structures of subjects' developmental exposure profiles to various elements. Fig 5 illustrates this process, with recurrence matrices shown for three different elements from a single subject in our discovery cohort. These exhibit notably different properties, with the element in Fig 5A yielding long diagonal recurrence structures characteristic of a periodic process, while the element in Fig 5B exhibited more laminar structures (Fig 5B; note block-like structures composed of vertical/horizontal lines) indicative of a stable system. Fig 5C, in contrast to each of these, exhibits only minimal periodic structural features and these emerge only very briefly. Table 1 summarizes quantification of these example elements via RQA. We confirmed the validity of these measures with an analysis of twin surrogates, in which each the sequential organization of each developmental profile was randomized to create a surrogate and each surrogate was analyzed with RQA. We consistently found significant differences between the original and randomized signals on *Mean Diagonal Length*, *Entropy*, *Determinism*, *Laminarity* and *Trapping Time*, where randomized signals were less periodic, persistent and complex (see Appendix C annotations in S1 File and Table 2). These results confirm that the recurrence structures quantified in the observed data are genuine features reflecting an underlying temporal organization rather than artifacts of autocorrelations.

**Table 1:** Measures derived from recurrence quantification analysis (RQA) of the recurrence plots shown in Fig 5.

	Ca	Mn	As
**Determinism**	0.84	0.89	0.79
**Mean Diagonal Length**	4.24	3.88	3.23
**Entropy**	1.91	1.85	1.52
**Laminarity**	0.30	0.58	0.31
**Trapping Time**	2.12	3.12	2.48
**Recurrence Time**	11.11	6.74	10.62

**Table 2:** Surrogate recurrence quantification analysis.

Chi Square Goodness of Fit			
	Zn	Ca	Pb
**Determinism**	<0.0001	0.0049	<0.0059
**Mean Diagonal Length**	<0.0001	<0.0001	0.0005
**Entropy**	<0.0001	<0.0001	0.001
**Laminarity**	<0.0001	<0.0001	<0.0001
**Trapping Time**	<0.0001	<0.0001	<0.0001
**Recurrence Time**	0.0004	ns	0.0035

**Fig 6:**
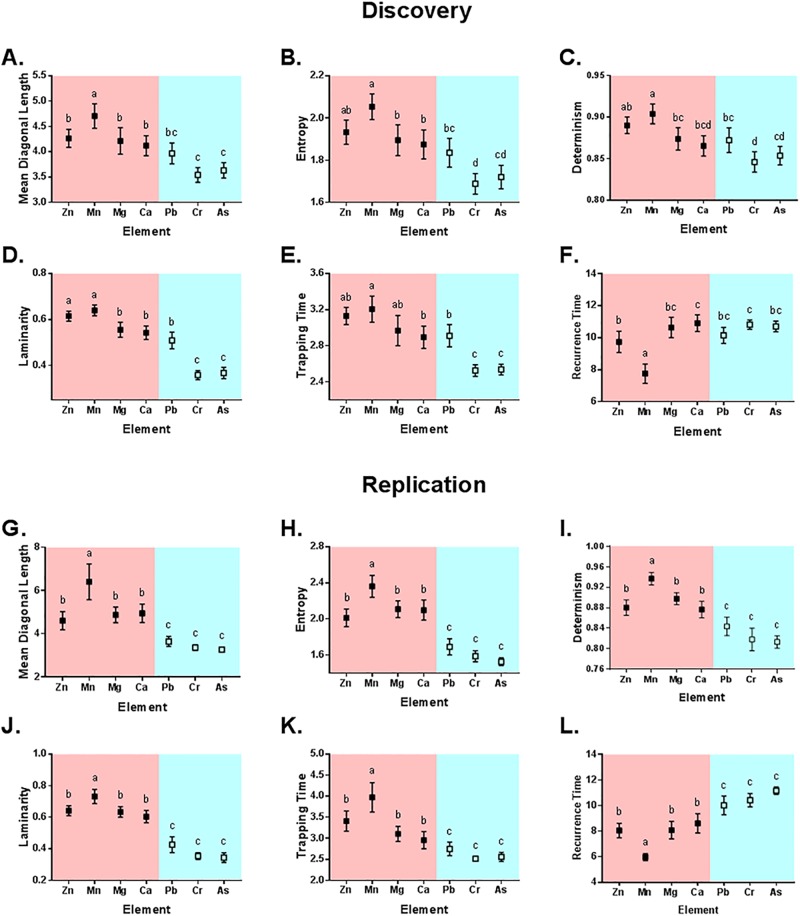
Comparison of discovery cohort and replication cohort recurrence measures in nutrient (red) and toxicant (blue) elements. Plots show the mean and 95% confidence intervals for Determinism (A, G), Mean Diagonal Length (B, H), Entropy (C, I), Laminarity (D, J), Trapping Time (E, K), and Recurrence Time (F, L). Means that have no superscript in common are significantly different (p < 0.05).

In our statistical analysis of periodic components in our discovery cohort’s elemental exposure profiles, we found that elemental type drove significant differences in *Determinism* (F(6,244) = 11.03, p <0.0001), *Mean Diagonal Length* (F(6,244) = 18.18, p < 0.0001), *Entropy* (F(6,244) = 19.03, p < 0.0001), and *Recurrence Time* (F(4,244) = 20.24, p < 0.0001), with periodic components more abundant, more persistent, exhibiting greater complexity, and with shorter intervals between periodic components in nutrient elements as compared to toxicants (i.e., the distributions in pink are higher than those in blue). Post-hoc analyses (see Fig 6 annotations and Appendix D in S1 File) confirmed these effects were generally attributable to reduced periodicity in Cr and As profiles relative to nutrients, while Pb generally followed the periodic signature of Ca exposure profiles. Interestingly, among nutrients Mn exhibited the strongest periodic signature, with greater determinism, diagonal length, and entropy than other nutrients. Additionally, the periodic properties of Mn signals were pronounced relative to other essential elements, exhibiting longer diagonal lengths, entropy, and determinism, with shorter intervals between cyclical processes (recurrence time). Sex was not a significant determinant of any periodic signatures.

We identified remarkably similar patterns in our analysis of periodic components in the elemental exposure profiles of our second replication cohort, and found again that elemental type drove significant differences in *Determinism* (F(6,144) = 33.89, p < 0.0001), *Mean Diagonal Length* (F(6,144) = 28.98, p < 0.0001), *Entropy* (F(6,144) = 49.54, p < 0.0001), and *Recurrence Time* (F(6,144) = 40.39, p < 0.0001), with higher measured values for nutrients compared to toxicants (see Fig 6 annotations, and Appendix E in S1 File). Furthermore, we found again that the dynamical properties of Mn were pronounced relative to other essential elements, exhibiting longer diagonal lengths, entropy, and determinism, and shorter recurrence time. Interestingly, we found reduced periodicity in all toxicants, including Pb, when compared to nutrient elements. As in our discovery cohort, sex had no significant effect on periodic signatures in the replication cohort.

We also analyzed signal stability in developmental exposure profiles, and found significant differences across elements in *Laminarity* (F(7,244) = 70.59, p<0.0001) and *Trapping Time* (F(7,244) = 22.41, p < 0.0001), with nutrients exhibiting greater signal stability and persisting in stable states for longer than toxicants. Again, in the discovery cohort, Pb proved an exception to this general pattern, with properties most closely aligned to Ca signals. Sex was not a significant determinant of *Laminarity* or *Trapping Time*. In the replication cohort, nutrients were also more stable than toxicants with higher values of *Laminarity* (F(6,144) = 73.53, p < 0.0001) and *Trapping Time* (F(6,144) = 25.68, p < 0.0001); however, we found reduced stability in all toxicants. Sex was also not a determinant on the persistence of elemental states in the replication cohort.

### Cross-recurrence quantification analysis

**Fig 7:**
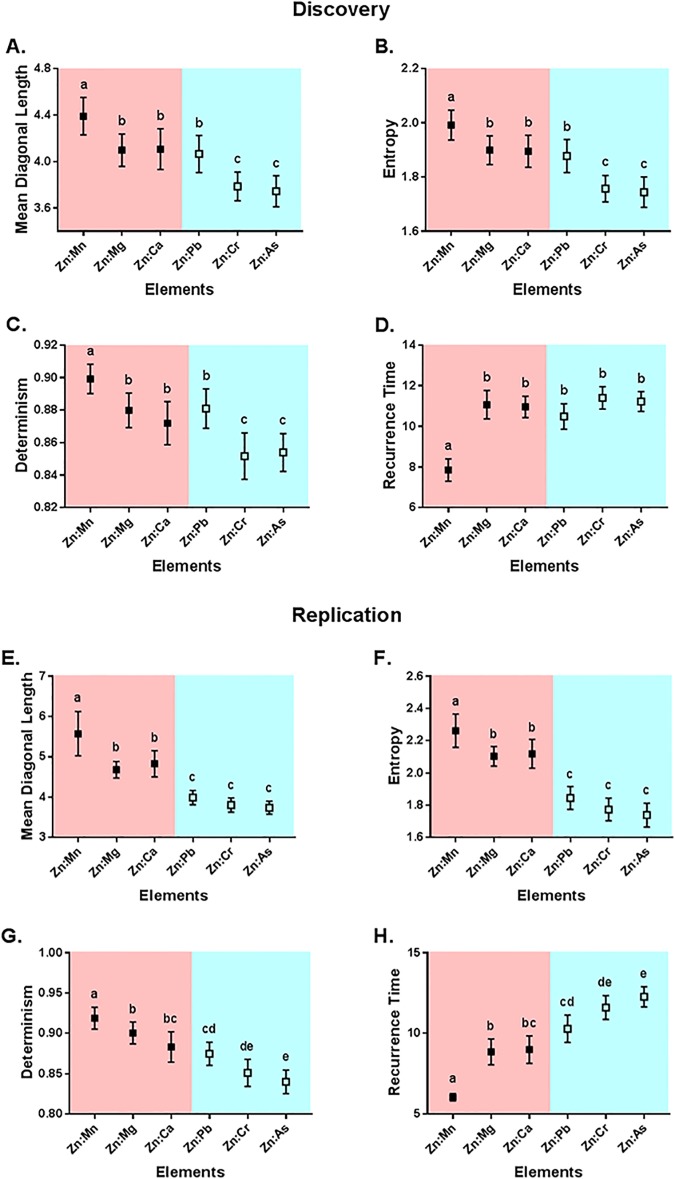
Cross-recurrence quantification analysis of nutrient and toxic elements. Plots show the mean and 95% confidence intervals in discovery (top panel) and replication (bottom panel) cohorts for Mean Diagonal Length (A, E), Entropy (B, F), Determinism (C, G), Recurrence Time (D, H). Means that have no superscript in common are significantly different (p < 0.05).

We next extended this technique to a multivariate analysis (CRQA) of periodic signal components, focusing on the synchronization of periodic components in Zn exposure profiles with other elements. In Fig 7 (top panel) we show the results of this CRQA in our discovery cohort, emphasizing again that synchronous dynamics among nutrients differ from toxicants. We confirmed statistically significant differences across Zn-based elemental cross-recurrences in *Determinism* (F(5,203) = 25.53, p< 0.0001), *Mean Diagonal Length* (F(5,203) = 24.95, p < 0.0001), *Entropy* (F(5,203) = 28.57, p < 0.0001), and *Recurrence Time* (F(5,203) = 25.63, p < 0.0001), with nutrient cross-recurrences exhibiting greater periodic structure, more persistent synchronization, and greater complexity than cross-recurrences with toxicants. Sex was not a significant determinant of these processes. Post-hoc analyses (see Fig 7 annotations and Appendix F in S1 File) confirmed a pattern consistent with analyses of individual elemental periodicities, in that Zn cross-recurrences with Cr and As differed from nutrients, but interactions with Pb continue to follow a signature consistent with Ca.

We further replicated the CRQA analysis in our replication cohort and confirmed the striking difference between nutrient and toxicant elemental profiles; the results are shown in Fig 7 (bottom panel). We found significant differences in Zn-focused CRQA across all measures, including *Determinism* (F(5,120) = 33.39, p < 0.0001), *Mean Diagonal Length* (F(5,120) = 39.56, p < 0.0001), *Entropy* (F(5,120) = 53.37, p < 0.0001), and *Recurrence Time* (F(5,120) = 40.35, p < 0.0001). Post-hoc analyses (Appendix G in S1 File) confirmed that toxicants have reduced temporal dynamic properties, including Pb. In contrast to our discovery set, we did not find a similar signature between Ca and Pb in our replication set.

## Discussion

Our results emphasize that the dynamic properties of elemental exposure profiles during development differ significantly across element types, and these differences, with some exceptions, generally partition broadly between nutrient elements and toxicant elements. We found these patterns remarkably consistent in two distinct cohorts representing populations in Sweden and the United States, which suggests an underlying commonality in the processes underlying elemental metabolism across different environments.

The dynamic properties of Zn, Mn, Mg, and Ca differed from those of Cr and As in every measure derived from recurrence quantification analysis (RQA). This pattern was again evident when we extended RQA to examine differences in cross recurrence focusing on Zn and other elements, with Zn:Cr and Zn:As cross-recurrence differing from cross recurrence involving Zn and other elements on multiple measures. Two notable exceptions to these patterns are discussed in greater depth in the following. First, in our discovery cohort we found that Pb, unlike other toxicant elements, generally did not differentiate from nutrients on most measures; and, second, that Mn, among elements with nutrient qualities, generally differed from other nutrients (as well as toxicants) in multiple dynamic properties.

The developmental exposure profiles of Pb and nutrient elements, particularly Ca, exhibited surprisingly similar dynamical properties, given the stark contrasts evident between nutrients and Cr and As. We suggest the framework of ‘ionic mimicry’ offers a plausible explanation for this observation [46]. Since Pb is a non-essential toxicant and lacks specific endogenous transporter systems, it is metabolized by Ca transporters due to its ionic similarity to Ca [46]. In teeth and other apatite containing matrices, Pb replaces the Ca ions from the hydroxyapatite lattice [47]. Supporting this interpretation, we found the dynamical properties of Pb exposures profiles were most like Ca profiles, suggesting the rise and ebb of Pb and Ca signals were driven by a common underlying mechanism. We did not, however, find that this pattern was reproduced in our replication cohort, where Pb tended to differ from other nutrients, though possibly the smaller sample size in our replication set occluded detection of this pattern.

In both our discovery and replication cohorts, we consistently found that the dynamic properties of Mn signals differed from other nutrients, including Zn, Mn, Mg, and Ca. This pattern was again apparent in cross-recurrence involving Zn and Mn, which yielded greater determinism, longer diagonal lengths, and greater entropy than in interactions with other nutrients. These distinctions may relate to the dynamic role of Mn in development where it acts within some developmental windows as an essential nutrient, but can be neurotoxic at other developmental periods [48].

We have presented the conceptual framework for RQA and CRQA, and demonstrated its potential utility in environmental epidemiologic studies by using these methods to contrast the dynamic properties inherent in the metabolism of essential elements and toxic elements. These results present a critical baseline characterization of cyclical features in the metabolism of exogenous elements, which appear broadly similar across different populations, and may serve as the impetus for future studies to examine the role of these processes in human health. In particular these methods lend themselves to epidemiological studies, where the dynamical processes that metabolize various chemicals and biomolecules can be contrasted between health outcomes. This will allow future studies to identify the role of periodic endogenous rhythms, and potentially their disruption, in the etiology of disease.

## Supporting information

S1 File Supporting information. Appendix A: Recurrence plot for noisy perioidic signal. Appendix B: Recurrence plot for elemental exposure profile. Appendix C: Analysis of surrogate time series data. Appendix D: Discovery-cohort post-hoc analyses of elemental RQA. Appendix E: Replication-cohort post-hoc analyses of elemental RQA. Appendix F: Discovery-cohort post-hoc analyses of elemental cross-recurrences. Appendix G: Replication-cohort post-hoc analyses of elemental cross-recurrences.
(DOCX)
