# Supplementary material for: Recurrence quantification analysis to characterize cyclical components of environmental elemental exposures during fetal and postnatal development
Source: PLoS One. 2017 Nov 7;12(11):e0187049. doi: 10.1371/journal.pone.0187049 (PMC5675384; doi:10.1371/journal.pone.0187049)
Supplement: S1 File — Appendix A: Recurrence plot for noisy perioidic signal. Appendix B: Recurrence plot for elemental exposure profile. Appendix C: Analysis of surrogate time series data. Appendix D: Discovery-cohort post-hoc analyses of elemental RQA. Appendix E: Replication-cohort post-hoc analyses of elemental RQA. Appendix F: Discovery-cohort post-hoc analyses of elemental cross-recurrences. Appendix G: Replication-cohort post-hoc analyses of elemental cross-recurrences. (DOCX) [file pone.0187049.s001.docx]

Supporting Information

Recurrence quantification analysis to characterize cyclical components of environmental elemental exposures during fetal and postnatal development

Paul Curtin^1¶*^, Austen Curtin^2¶^, Christine Austin^2^, Chris Gennings^1^, Kristiina Tammimies^3,4^, Sven Bölte^3,4^, Manish Arora^2*^

^1^Department of Environmental Medicine and Public Health, Icahn School of Medicine at Mount Sinai, New York City, New York, USA.

^2^Senator Frank R Lautenberg Environmental Health Sciences Laboratory, Department of Environmental Medicine and Public Health, Division of Environmental Health, Icahn School of Medicine at Mount Sinai, New York City, New York, USA

^3^Department of Women’s and Children’s Health, Center of Neurodevelopmental Disorders (KIND), Karolinska Institutet, Stockholm, Sweden

^4^Center for Psychiatry Research, Stockholm County Council, Stockholm, Sweden

Corresponding authors

E-mail: [manish.arora@mssm.edu](mailto:manish.arora@mssm.edu) (MA)

E-mail: [paul.curtin@mssm.edu](mailto:paul.curtin@mssm.edu) (PC)

^¶^These authors contributed equally to this manuscript.

**Appendix A. Recurrence plot for noisy perioidic signal.**

**Appendix B. Recurrence plot for elemental exposure profile**.

**Appendix C. Analysis of surrogate time series data.**

**Appendix D. Discovery-cohort post-hoc analyses of elemental RQA**.

**Appendix E. Replication-cohort post-hoc analyses of elemental RQA.**

**Appendix F. Discovery-cohort post-hoc analyses of elemental cross-recurrences**.

**Appendix G. Replication-cohort post-hoc analyses of elemental cross-recurrences.**

**Appendix A**

**
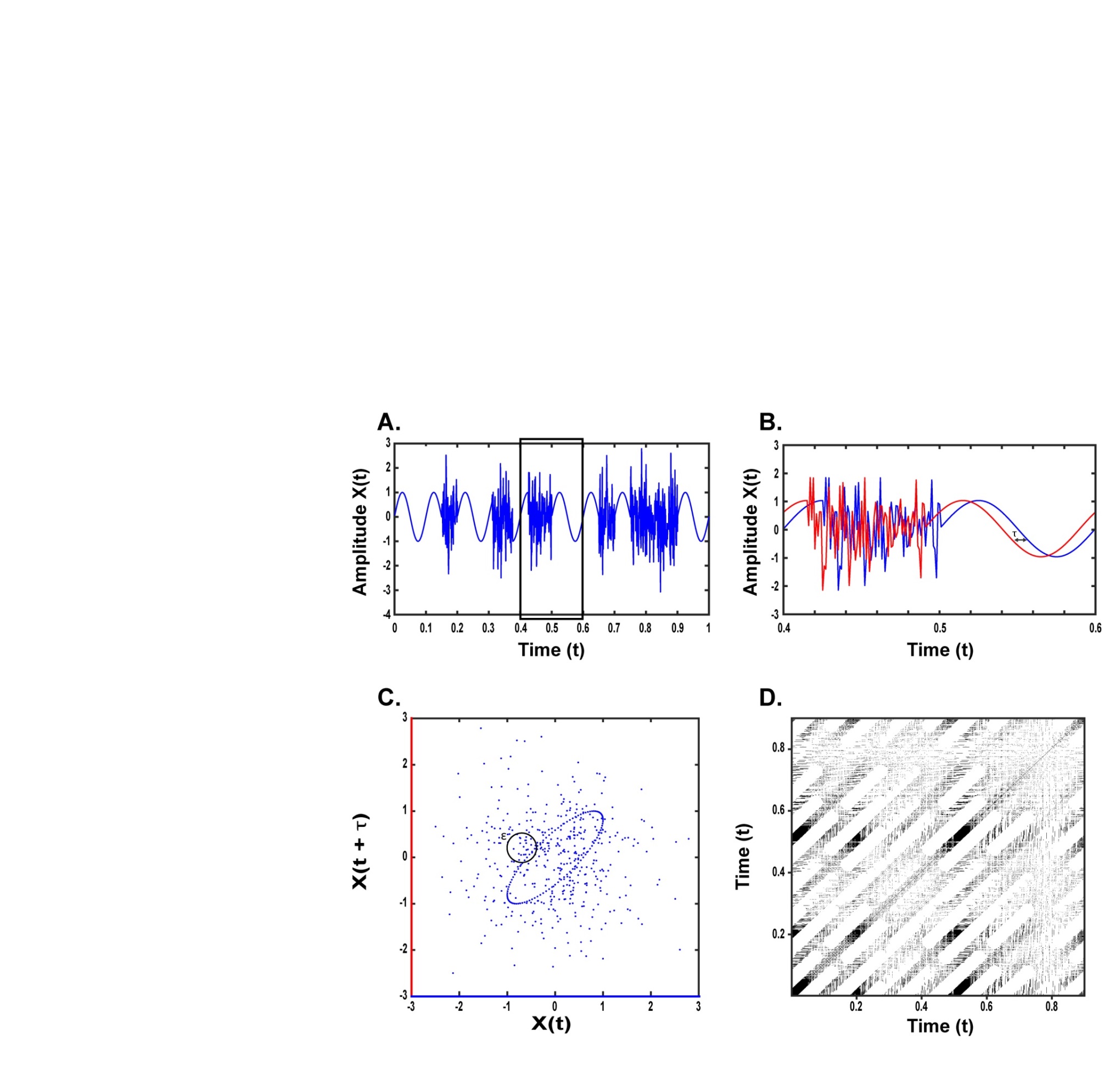
**

**Recurrence plot for noisy perioidic signal.** A) A 10 Hz sinusoid with intermittent random noise. B) In blue, 200 ms of the signal shown in A (indicated by the gray box in panel). Red line is a vector generated by a delay embedding, whereby the original signal is duplicated with a time-lagged delay of interval (*τ*), here 10 ms. C) Phase-portrait of the 10 Hz sinusoid in (A) plotted in two-dimensions (see text). The x-y coordinates of a given point are determined by the value of amplitude of the delayed embedded signals at each sampling interval. Black circle indicates an exemplar threshold (*ε*) used to construct recurrence matrix (D). D) Recurrence plot of signal in A.

**Appendix B**

**
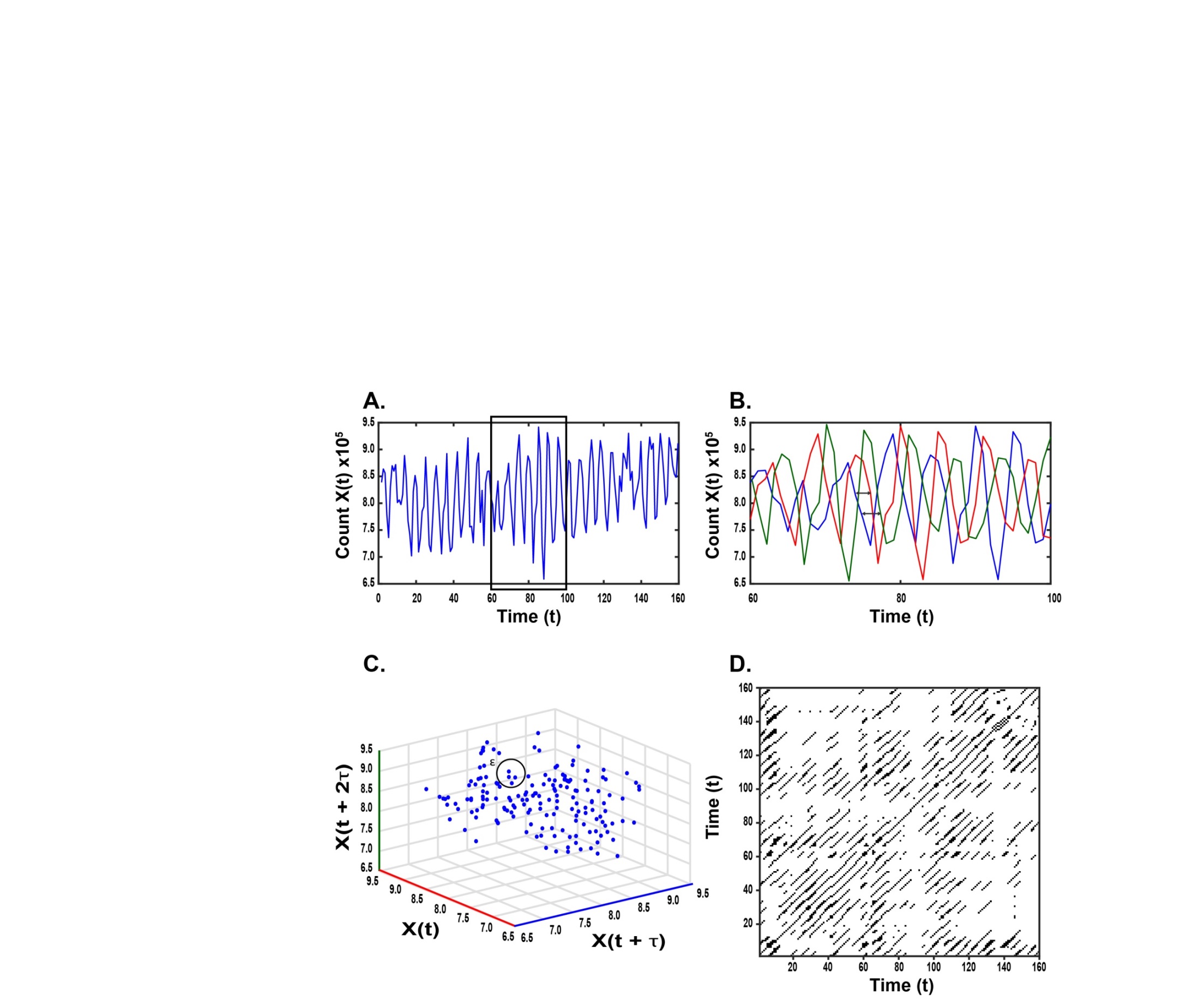
Recurrence plot for elemental exposure profile**. A) Developmental exposure profile for Ca, expressed as count over time (days), reflecting elemental concentrations in tooth matrices from -89 to 300 days since birth. B) Delay embedding of the signal in (A), showing the paneled-section of the waveform. The original signal is shown in blue, with green and red lines reflecting two embeddings (time-lagged duplicates) of delay (*τ*) 10. C) Phase-portrait of the Ca time-series in (A) plotted in three-dimensions. The coordinates of a given point are determined by the value of each delay-embedded signal at that time interval. Black circle indicates an exemplar threshold (ε) used to construct a recurrence matrix (D). D) Recurrence plot of the Ca time-series. Black marks in matrix indicate time-points where the system entered a given state (within-threshold on phase-portrait). Diagonal structures in this plot reflect periods of stable periodic orbits, while white space indicates unique values, and laminar structures (vertical/horizontal lines) reflect static states. During recurrence quantification analysis (RQA), the duration and distribution of periodic, singular, and laminar states are quantified.

**Appendix C**

**
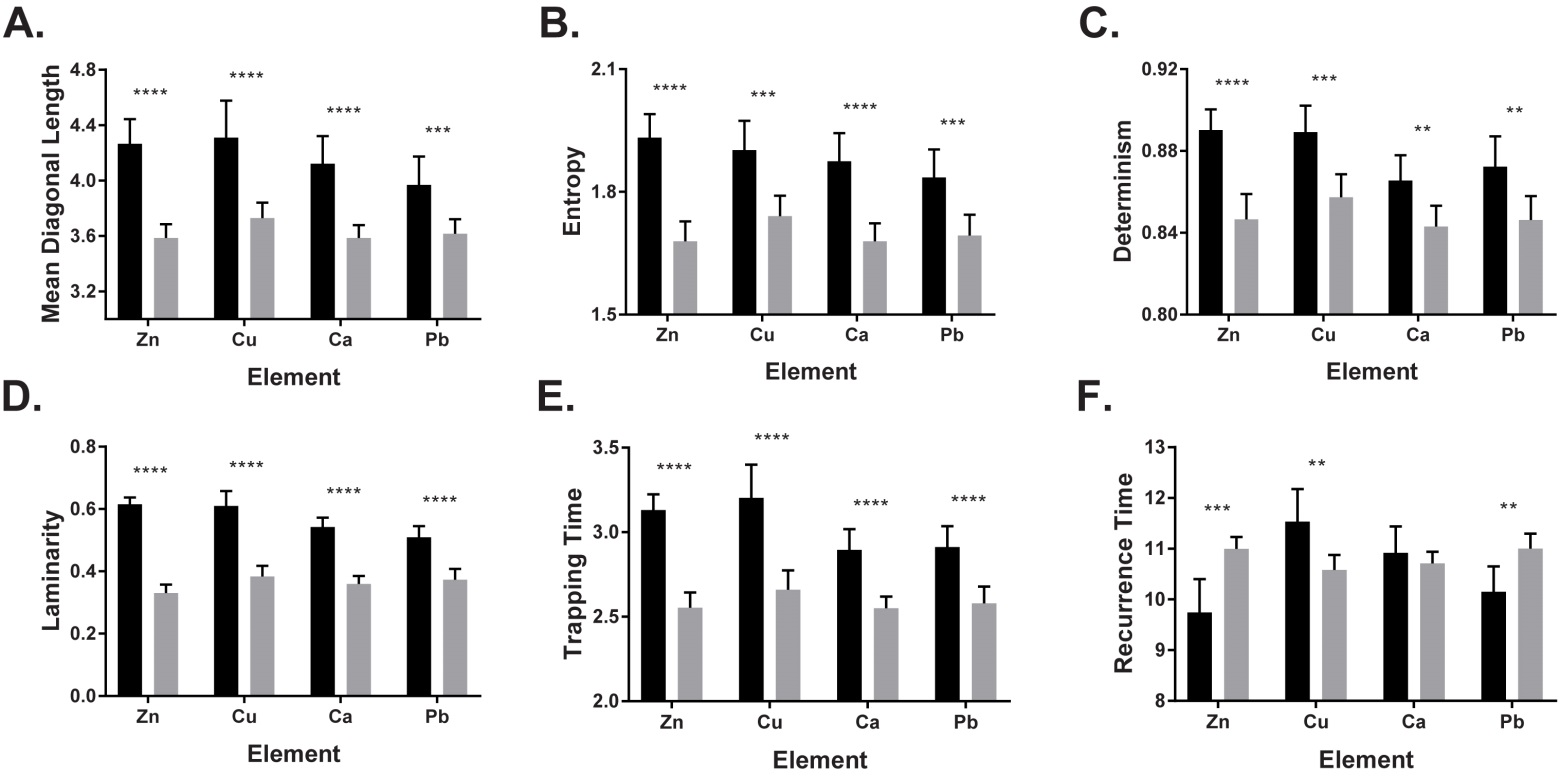
**

**Analysis of surrogate time series data.** Comparison of recurrence analyses on raw element concentration traces (black bars) to randomized surrogate traces (gray bars).

**Appendix D**

**Discovery-cohort post-hoc analyses of elemental RQA**.

| **Tukey-Kramer Adjusted P-values** | | | | | | | |
| --- | --- | --- | --- | --- | --- | --- | --- |
| **Element 1** | **Element 2** | **Determinism** | **Mean Diagonal Length** | **Entropy** | **Laminarity** | **Trapping Time** | **Recurrence Time** |
| **As** | **Ca** | ns | *0.0047* | *0.0032* | *<.0001* | *0.0002* | ns |
| **As** | **Cr** | ns | ns | ns | ns | ns | ns |
| **As** | **Mg** | ns | *0.0003* | *0.0005* | *<.0001* | *<.0001* | ns |
| **As** | **Mn** | *<.0001* | *<.0001* | *<.0001* | *<.0001* | *<.0001* | *<.0001* |
| **As** | **Pb** | ns | ns | ns | *<.0001* | *<.0001* | ns |
| **As** | **Zn** | *0.0005* | *<.0001* | *<.0001* | *<.0001* | *<.0001* | ns |
| **Ca** | **Cr** | ns | *0.0003* | *0.0001* | *<.0001* | *<.0001* | ns |
| **Ca** | **Mg** | ns | ns | ns | ns | ns | ns |
| **Ca** | **Mn** | *0.0002* | *0.0003* | *0.0003* | *<.0001* | *0.0021* | *<.0001* |
| **Ca** | **Pb** | ns | ns | ns | ns | ns | ns |
| **Ca** | **Zn** | ns | ns | ns | *0.0027* | ns | *0.0161* |
| **Cr** | **Mg** | *0.0218* | *<.0001* | *<.0001* | *<.0001* | *<.0001* | ns |
| **Cr** | **Mn** | *<.0001* | *<.0001* | *<.0001* | *<.0001* | *<.0001* | *<.0001* |
| **Cr** | **Pb** | *0.0403* | *0.0263* | *0.0079* | *<.0001* | *<.0001* | ns |
| **Cr** | **Zn** | *<.0001* | *<.0001* | *<.0001* | *<.0001* | *<.0001* | *0.0376* |
| **Mg** | **Mn** | *0.0088* | *0.0043* | *0.0023* | *0.0002* | ns | *<.0001* |
| **Mg** | **Pb** | ns | ns | ns | ns | ns | ns |
| **Mg** | **Zn** | ns | ns | ns | *0.0281* | ns | ns |
| **Mn** | **Pb** | *0.0058* | *<.0001* | *<.0001* | *<.0001* | *0.0056* | *<.0001* |
| **Mn** | **Zn** | ns | *0.0162* | ns | ns | ns | *<.0001* |
| **Pb** | **Zn** | ns | ns | ns | *<.0001* | ns | ns |

Post hoc comparisons among elements (columns 1 and 2) for periodic properties derived from RQA. Values given are p-values adjusted for multiple comparisons (Tukey).

**Appendix E**

**Replication-cohort post-hoc analyses of elemental RQA.**

| **Tukey-Kramer Adjusted P-values** | | | | | | | |
| --- | --- | --- | --- | --- | --- | --- | --- |
| **Element 1** | **Element 2** | **Determinism** | **Mean Diagonal Length** | **Entropy** | **Laminarity** | **Trapping Time** | **Recurrence Time** |
| **As** | **Ca** | *<.0001* | *<.0001* | *<.0001* | *<.0001* | ns | *<.0001* |
| **As** | **Cr** | ns | ns | ns | ns | ns | ns |
| **As** | **Mg** | *<.0001* | *<.0001* | *<.0001* | *<.0001* | *0.0041* | *<.0001* |
| **As** | **Mn** | *<.0001* | *<.0001* | *<.0001* | *<.0001* | *<.0001* | *<.0001* |
| **As** | **Pb** | ns | ns | ns | *0.0281* | ns | ns |
| **As** | **Zn** | *<.0001* | *0.0002* | *<.0001* | *<.0001* | *<.0001* | *<.0001* |
| **Ca** | **Cr** | *<.0001* | *<.0001* | *<.0001* | *<.0001* | *0.043* | *0.0002* |
| **Ca** | **Mg** | ns | ns | ns | ns | ns | ns |
| **Ca** | **Mn** | *<.0001* | *<.0001* | *0.001* | *<.0001* | *<.0001* | *<.0001* |
| **Ca** | **Pb** | *0.0431* | *0.0004* | *<.0001* | *<.0001* | ns | *0.0089* |
| **Ca** | **Zn** | ns | ns | ns | ns | *0.0328* | ns |
| **Cr** | **Mg** | *<.0001* | *<.0001* | *<.0001* | *<.0001* | *0.0014* | *<.0001* |
| **Cr** | **Mn** | *<.0001* | *<.0001* | *<.0001* | *<.0001* | *<.0001* | *<.0001* |
| **Cr** | **Pb** | ns | ns | ns | ns | ns | ns |
| **Cr** | **Zn** | *<.0001* | *0.0008* | *<.0001* | *<.0001* | *<.0001* | *<.0001* |
| **Mg** | **Mn** | *0.0074* | *<.0001* | *0.0017* | *0.0036* | *<.0001* | *<.0001* |
| **Mg** | **Pb** | *<.0001* | *0.001* | *<.0001* | *<.0001* | ns | *<.0001* |
| **Mg** | **Zn** | ns | ns | ns | ns | ns | ns |
| **Mn** | **Pb** | *<.0001* | *<.0001* | *<.0001* | *<.0001* | *<.0001* | *<.0001* |
| **Mn** | **Zn** | *<.0001* | *<.0001* | *<.0001* | *0.011* | *0.0028* | *<.0001* |
| **Pb** | **Zn** | *0.0153* | *0.022* | *<.0001* | *<.0001* | *0.0002* | *<.0001* |

Post hoc comparisons among elements (columns 1 and 2) for periodic properties derived from RQA. Values given are p-values adjusted for multiple comparisons (Tukey).

**Appdendix F**

**Discovery-cohort post-hoc analyses of elemental cross-recurrences**.

| **Tukey-Kramer Adjusted P-values** | | | | | |
| --- | --- | --- | --- | --- | --- |
| **Element 1** | **Element 2** | **Determinism** | **Mean Diagonal Length** | **Entropy** | **Recurrence Time** |
| **Zn-As** | **Zn-Ca** | *0.0056* | *<.0001* | *<.0001* | ns |
| **Zn-As** | **Zn-Cr** | ns | ns | ns | ns |
| **Zn-As** | **Zn-Mg** | *<.0001* | *<.0001* | *<.0001* | ns |
| **Zn-As** | **Zn-Mn** | *<.0001* | *<.0001* | *<.0001* | *<.0001* |
| **Zn-As** | **Zn-Pb** | *<.0001* | *<.0001* | *<.0001* | ns |
| **Zn-Ca** | **Zn-Cr** | *0.001* | *<.0001* | *<.0001* | ns |
| **Zn-Ca** | **Zn-Mg** | ns | ns | ns | ns |
| **Zn-Ca** | **Zn-Mn** | *<.0001* | *0.0005* | *0.0019* | *<.0001* |
| **Zn-Ca** | **Zn-Pb** | ns | ns | ns | ns |
| **Zn-Cr** | **Zn-Mg** | *<.0001* | *<.0001* | *<.0001* | ns |
| **Zn-Cr** | **Zn-Mn** | *<.0001* | *<.0001* | *<.0001* | *<.0001* |
| **Zn-Cr** | **Zn-Pb** | *<.0001* | *0.0012* | *<.0001* | ns |
| **Zn-Mg** | **Zn-Mn** | *0.0022* | *0.0003* | *0.0035* | *<.0001* |
| **Zn-Mg** | **Zn-Pb** | ns | ns | ns | ns |
| **Zn-Mn** | **Zn-Pb** | *0.0041* | *<.0001* | *0.0001* | *<.0001* |

Post-hoc comparisons among cross recurrences (columns 1 and 2) for various periodic properties derived from CRQA. Numbers given are p values adjusted for multiple comparisons (Tukey).

**Appendix G**

**Replication-cohort post-hoc analyses of elemental cross-recurrences.**

| **Tukey-Kramer Adjusted P-values** | | | | | |
| --- | --- | --- | --- | --- | --- |
| **Element 1** | **Element 2** | **Determinism** | **Mean Diagonal Length** | **Entropy** | **Recurrence Time** |
| **Zn-As** | **Zn-Ca** | *<.0001* | *<.0001* | *<.0001* | *<.0001* |
| **Zn-As** | **Zn-Cr** | ns | ns | ns | ns |
| **Zn-As** | **Zn-Mg** | *<.0001* | *<.0001* | *<.0001* | *<.0001* |
| **Zn-As** | **Zn-Mn** | *<.0001* | *<.0001* | *<.0001* | *<.0001* |
| **Zn-As** | **Zn-Pb** | *0.0006* | ns | ns | 0.0062 |
| **Zn-Ca** | **Zn-Cr** | *0.0003* | *<.0001* | *<.0001* | *<.0001* |
| **Zn-Ca** | **Zn-Mg** | ns | ns | ns | ns |
| **Zn-Ca** | **Zn-Mn** | *<.0001* | *0.0002* | *0.0124* | *<.0001* |
| **Zn-Ca** | **Zn-Pb** | ns | *<.0001* | *<.0001* | ns |
| **Zn-Cr** | **Zn-Mg** | *<.0001* | *<.0001* | *<.0001* | *<.0001* |
| **Zn-Cr** | **Zn-Mn** | *<.0001* | *<.0001* | *<.0001* | *<.0001* |
| **Zn-Cr** | **Zn-Pb** | ns | ns | ns | ns |
| **Zn-Mg** | **Zn-Mn** | ns | *<.0001* | *0.0036* | *<.0001* |
| **Zn-Mg** | **Zn-Pb** | *0.0011* | *0.0002* | *<.0001* | *0.0205* |
| **Zn-Mn** | **Zn-Pb** | *<.0001* | *<.0001* | *<.0001* | *<.0001* |

Post-hoc comparisons among cross recurrences (columns 1 and 2) for various periodic properties derived from CRQA. Numbers given are p values adjusted for multiple comparisons (Tukey).
